# Child-Staff Ratios in Early Childhood Education and Care Settings and Child Outcomes: A Systematic Review and Meta-Analysis

**DOI:** 10.1371/journal.pone.0170256

**Published:** 2017-01-19

**Authors:** Michal Perlman, Brooke Fletcher, Olesya Falenchuk, Ashley Brunsek, Evelyn McMullen, Prakesh S. Shah

**Affiliations:** 1 Applied Psychology and Human Development, University of Toronto/OISE, Toronto, Ontario, Canada; 2 Department of Gastroenterology, Alberta Children's Hospital, Calgary, Alberta, Canada; 3 Department of Pediatrics, Mount Sinai Hospital, Toronto, Ontario, Canada; 4 Department of Pediatrics, University of Toronto, Toronto, Ontario, Canada; 5 Institute of Health Policy, Management and Evaluation, University of Toronto, Toronto, Ontario, Canada; TNO, NETHERLANDS

## Abstract

Child-staff ratios are a key quality indicator in early childhood education and care (ECEC) programs. Better ratios are believed to improve child outcomes by increasing opportunities for individual interactions and educational instruction from staff. The purpose of this systematic review, and where possible, meta-analysis, was to evaluate the association between child-staff ratios in preschool ECEC programs and children’s outcomes. Searches of Medline, PsycINFO, ERIC, websites of large datasets and reference sections of all retrieved articles were conducted up to July 3, 2015. Cross-sectional or longitudinal studies that evaluated the relationship between child-staff ratios in ECEC classrooms serving preschool aged children and child outcomes were independently identified by two reviewers. Data were independently extracted from included studies by two raters and differences between raters were resolved by consensus. Searches revealed 29 eligible studies (31 samples). Child-staff ratios ranged from 5 to 14.5 preschool-aged children per adult with a mean of 8.65. All 29 studies were included in the systematic review. However, the only meta-analysis that could be conducted was based on three studies that explored associations between ratios and children’s receptive language. Results of this meta-analysis were not significant. Results of the qualitative systematic review revealed few significant relationships between child-staff ratios and child outcomes construed broadly. Thus, the available literature reveal few, if any, relationships between child-staff ratios in preschool ECEC programs and children’s developmental outcomes. Substantial heterogeneity in the assessment of ratios, outcomes measured, and statistics used to capture associations limited quantitative synthesis. Other methodological limitations of the research integrated in this synthesis are discussed.

## Introduction

Early childhood is a critical period in shaping children’s developmental trajectories [1]. Research has shown that high quality early childhood education and care (ECEC) programs can enhance child development [2,3]. Given that enrollment in ECEC settings has become the norm for preschool aged children [4,5], it is important to empirically identify aspects of the ECEC environment that are key in supporting children’s development. Organizations such as the American Academy of Pediatrics (AAP), the American Public Health Association (APHA), the National Institute of Early Education Research (NIEER), and the National Association for the Education of Young Children (NAEYC) have identified indicators of high quality ECEC programs. These guidelines are intended to assist parents, policy makers, funders and other key stakeholders in making informed decisions about ECEC arrangements. The ratio of children to staff is one relatively quantifiable aspect of *structural* quality identified as a key quality indicator in the AAP’s Policy Statement on Quality Early Education and Child Care [6–10].

The perceived importance of low child-staff ratios in ECEC programs is demonstrated by the fact that all Quality Rating and Improvement Systems (QRIS) in North America include child-staff ratios in their ratings of quality [11]. QRIS are accountability systems that revolve around multicomponent assessments designed to improve the quality of ECEC programs. Regulations regarding child-staff ratios in licensed ECEC settings are implemented at various levels of government in the U.S (Table 1 for a summary of ratio recommendations); however, there is wide variability between jurisdictions with few jurisdictions in the US and Canada meeting these recommended standards [12,13].

**Table 1:** Recommended Standard for Child-Staff Ratios in Center-Based ECEC Settings for Preschool-Aged Classrooms.

Advisory Group	Age Group			
	2 years	3 years	4 years	5 years
**AAP, 2005**	*24–30 months*:			
	4:1 with group size < 8	7:1 with group size < 14	8:1 with group size < 16	8:1 with group size < 16
	*31–35 months*:			
	5:1 with group size <10			
**NAEYC, 2014**	*21–36 months*:	*30–48 months*:		
	4:1 for group size < 8	6:1 for group size < 12	8:1 for group size < 16	8:1 for group size < 16
	5:1 / < 10	7:1 / < 14	9:1 / < 18	9:1 / < 18
	6:1 / < 12	8:1 / < 16	10:1 / < 20	10:1 / < 20
		9:1 / < 18		
**UNICEF Innocenti Report Card, 2008**	N/A	N/A	15:1 with group size < 24	15:1 with group size < 24

Research findings on associations between child-staff ratios and outcomes have been contradictory. Some studies show that better ratios are associated with improved child outcomes [14–16] while other studies have not found such linkages [17–20] or report mixed results [21]. Many practitioners working with children and families are in a position to provide parents with information about ECEC for their children. By way of just one example of this, in its policy statement on quality early education and child care, the AAP notes that pediatricians have a significant role to play in helping parents identify high quality ECEC environments for their young children [10]. However, to do this effectively, practitioners must have access to evidence-based recommendations about which aspects of the ECEC environment are important. To our knowledge, a synthesis of available literature to identify appropriate or ideal ratios in relation to children’s outcomes has not been conducted. To provide such guidance, our objective in this study was to systematically review and, if possible, meta-analyze associations between child-staff ratios in ECEC classrooms that serve preschool-aged children and child outcomes.

## Method

### Types of Participants and Setting

This study focused on classrooms that serve preschool-aged children as these serve the largest number of children in ECEC settings [22]. Preschool-age was defined as ranging from 30 to 72 months. ECEC settings included child care centers, preschool programs, nursery schools, pre-kindergarten programs, and Head Start programs. Studies that only examined home-based child care, or those in which results for home-based and center-based care could not be separated were excluded.

### Types of Studies

Studies reporting associations in cohort, cross-sectional or longitudinal analyses were included in this review. Studies reporting a statistical link between an aggregate ECEC quality variable that consisted of several measures of quality and child outcomes were not included if the specific effect of ratios could not be separated. Case-series, reviews, editorials and letters to editors were read to identify articles but were not included in the review.

### Outcomes

Outcomes were operationalized broadly and included measures of children’s cognitive, pre-academic, social, emotional, behavioral, and motor functioning. Outcome measures included data that were gathered from direct testing of children as well as teacher and parent reports. Measures that focused on dyads (e.g., staff-child attachment) were excluded because it is difficult to separate “caregiver/program” effects from child characteristics using such measures.

### Search Strategy

An extensive search of the electronic databases PsycINFO, Medline, and ERIC was conducted for English language studies published until July 3, 2015. Two separate searches were performed within each of the three databases. One search combined search terms specific to child-staff ratios AND child outcomes. The other combined search terms related to more global ECEC quality indicators AND child outcomes. This increased the likelihood that we would capture studies in which ratios were used as a control variable. Specific keywords used in electronic searches are provided in Tables A-D in S1 File.

Second, websites of the Cost, Quality, and Outcomes Study (CQO) [23], Early Childhood Longitudinal Study (ECLS) [24]; Effective Provision of Pre-School Education (EPPE) Project [25]; Head Start Impact Study (HS) [26]; National Center for Early Development and Learning’s (NCEDL) Multi-State Study of Pre-Kindergarten and State-Wide Early Education Program Study (SWEEP) [27]; Head Start’s Family and Child Experiences Survey (FACES) [28] and the National Institute of Child Health and Human Development’s (NICHD) [29] Study of Early Child Care and Youth Development were reviewed to retrieve relevant studies. Lastly, reference lists of those studies that met inclusion criteria were manually searched to identify additional relevant studies (see Table 2 for a complete list of all inclusion criteria).

**Table 2:** Inclusion Criteria for Systematic Review and Rationale.

Criteria	Rationale
***Child Care Type***	
Only studies that examined the impact of the quality of centre-based programs on children’s outcomes were included. Centre-based programs included daycare and preschool programs, nursery schools, pre-kindergarten programs, and Head Start programs. Studies that only examined home-based child care, or those in which home-based and centre-based could not be separated were excluded.	Center-based child care settings differ from home daycare in many ways such as ratios, group size, physical environment, curriculum, age range of children, and caregiver qualifications. As a result, quality is often measured differently for these two settings (e.g., ECERS versus FCCERS).
***Age Served***	
Studies that included preschool-aged children as the majority of participants were included. For the purposes of the meta-analysis, preschool-age was defined as ranging from 30 to 72 months.	Preschool-aged classrooms are different from infant/toddler classrooms due to the developmental stage and needs of the children in these two age groups. As a result, regulations and standards of care (e.g., ratios, physical environment, etc.) as well as daily activities (e.g., curriculum) differ between infant/toddler and preschool-aged classrooms.
***Child Outcomes***	
Studies that provided information about the association between child-staff ratios on children’s cognitive, academic, social-emotional, health, or motor outcomes were included. Data could have been gathered from teachers, parents, and/or children themselves. Measures that focus on dyads (e.g., attachment) were excluded.	Cognitive, academic, social-emotional, health, and motor outcomes were selected because they are key predictors of children’s developmental trajectories. Measures that focus on child-staff or peer dyads were not included given that these outcomes often reflect an aspect of child care quality.
***Study Design***	
Cross-sectional and longitudinal designs were included. When multiple child outcome assessments were reported the earliest time-point following the measurement of quality were extracted.	To increase the homogeneity across the extracted data from eligible studies (i.e., increase the likelihood of meta-analysis), we focused on the earliest time-point in which child outcomes were measured following the measurement of quality in instances where multiple waves of outcome data were presented.
***Outcome Reporting***	
Studies must have presented statistical data quantifying the association between child-staff ratios and a child outcome measure.	Studies only reporting qualitative results were not considered for this review as the domains of assessment could vary markedly between studies.
***Language***	
To be extracted studies had to be in English.	We did not have resources to systematically translate material written in other languages.

Abbreviations: ECERS = Early Childhood Environment Rating Scale; FCCERS Family Child Care Environment Rating Scale.

### Selection Strategy

The title and abstract of each paper located through the literature searches was reviewed for relevance. Abstracts identified as potentially relevant to the current study underwent full-text review to determine if inclusion criteria were met (Table 2). Each step was conducted by a pair of independent raters.

### Data Extraction

Information was extracted from each study by two independent raters and then compared for accuracy. Discrepancies were resolved through discussion. If consensus could not be reached, the third author was consulted to make the final decision. Since raters extracted objective information (e.g., correlation statistics, descriptive statistics) disagreements were very rare.

### Meta Analyses

Studies that could be meta-analyzed were selected from the pool of studies selected for the systematic review. To be meta-analyzed studies had to use identical child outcome measures and had to use an identical operationalization of ratios. We adopted a minimum requirement of three studies to conduct a meta-analysis on a particular child outcome. To increase homogeneity among studies that were meta-analyzed, only studies that ensured children’s exposure to the program were included. Thus, we only meta-analyzed studies that used child pre-scores as a covariate, or used gain scores in analyses, or in which the authors stated explicitly that only children who had been in the program for a period of time prior to their assessment were included. In addition, only statistics that accounted for covariates (e.g., child and family characteristics) were combined within a single meta-analysis. Finally, to avoid overlap in samples where subsamples were drawn from the same dataset, only the study with the largest sample size was selected for inclusion in meta-analyses. Thus, within a given meta-analysis, only one coefficient from each sample was included in any one analysis.

Statistical models with quadratic terms assume non-linear associations between the variables. Given the statistics extracted for most studies only test for linear relationships (correlation coefficients and linear regression coefficients), associations in models using quadratic terms were excluded and only results examining linear relationships were used in the meta-analyses. We used random-effects models for meta-analyses. All meta-analyses were conducted using the Comprehensive Meta-Analysis Version 3 statistical package (see S2 File for formulas for converting statistics) [30]. Finally, for each meta-analysis *I*^*2*^ was used to test statistical heterogeneity.

## Results

**Fig 1:**
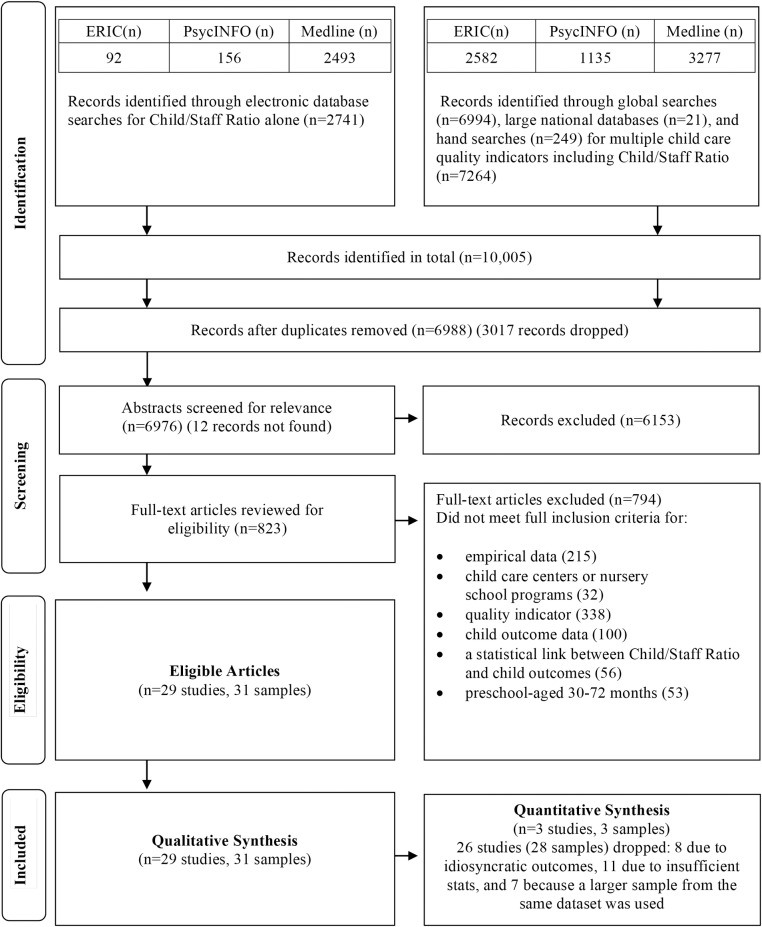
Flow diagram for study selection. Adapted from Moher et al.[46].

The details of the search results and reasons for exclusion of articles are presented in Fig 1. In total, 29 studies met our inclusion criteria; 23 of the studies were peer reviewed journal articles, five were reports and one study reported their results in a book chapter. Descriptive information for the 29 studies is presented in Table 3. Seven studies contained samples that were drawn from both the NCEDL’s Multi-State Study and SWEEP study [20,31–36], three studies were from the CQO project [21,37,38], two studies utilized the Head Start FACES 2000 Cohort sample [39,40], and two studies included samples drawn from the NICHD Study of Early Child Care [15,41]. Furthermore, two studies [42,43] were drawn from the same dataset as were two additional studies using a separate sample of preschool-aged children from Bermuda [44,45]. Thus, many studies had overlapping samples.

**Table 3:** Description of Studies Meeting Inclusion Criteria^a^.

Study^b^	Characteristics	Measurement of Child-Staff Ratios M(SD)^c^	Child Outcome Measures and Subscales M(SD)^d^	Statistics and Covariates^e^
Anders 2012[47]	• **Publication:** Journal (ECRQ) • **Design:** Longitudinal • **Dataset:** BiKs 3–10 Study • **Country:** Germany • **Sample size:** class 97; child 532 • **% Female:** 48.12 • **Mean age:** 37 • **Ethnicity:** 100% German • **Mean maternal education:** NR • **Mean household income:** NR	• 10.67 (2.70)	• K ABC-Arithmetic 15.08 (3.74)	• **Statistics Extracted:** B, SE • **Covariates:** pretest score, age, gender, mother's and father's language (German/other), SES, maternal education, age at entry to preschool, HLE-literacy, HLE-numeracy, interaction terms
Burchinal, Nelson 2000[38]^D^	• **Publication:** Journal (ECRQ) • **Design:** Longitudinal • **Dataset:** CQO Study • **Country:** United States (National) • **Sample size:** class NR; child 757 • **% Female:** 48.9 • **Mean Age:** 48.4 months • **Ethnicity:** C68%, B16%, H5%, O12% • **Mean maternal education:** 14.2 years • **Mean household income:** 24–72 K	• 7.72 (3.59)	• PPVT-R 93.59 (18.48)	• **Statistics Extracted:** B, SE • **Covariates:** gender, ethnicity, state, maternal education, CIS, teachers' responsiveness, child centered, group size. teacher education, ECERS
Burchinal, Roberts 2000[14]^T^	• **Publication:** Journal (CD) • **Design:** Longitudinal • **Dataset:** Otitis Media Study • **Country**: United States (South East) • **Sample size:** class 22; child 51 • **% Female:** 53% • **Mean age:** 36 months • **Ethnicity:** B100% • **Mean maternal education:** 12.5 years • **Mean household income:** 69% of sample were from households with income less than 185% of federal poverty threshold	• 9.00 (4.1)	• Bayley-MDI 95.74 (10.15) • SICD-RCA 33.44 (4.63) • SICD-ECA 35.36 (4.53)	• **Statistics Extracted:** Pearson’s Correlation • **Covariates:** none
Burchinal 2002[37]^m^^,^^D^	• **Publication:** Journal (ADS) • **Design:** Longitudinal • **Dataset:** CQO • **Country:** United States (National) • **Sample size:** class 418; child 828 • **% Female:** 48 • **Mean age:** 48.4 months • **Ethnicity:** C68%, B15%, A4%, H6%, O9% • **Mean maternal education:** NR • **Mean household income:** NR	• 8.02 (3.79)	PPVT-R (NR)	• **Statistics Extracted:** B, SE • **Covariates:** child/family level—gender, ethnicity, maternal education; classroom level–state, experience, teacher professional development (In-service workshops, workshops in the community, or workshops at professional meeting), formal training BA or BS in ECE or (a) AA/CDA in ECE, (b) college ECE courses, (c) workshops only/no formal training
Clarke-Stewart 1994[17]	• **Publication:** Book • **Design:** Longitudinal • **Country:** United States (Chicago) • **Sample size:** class NR; child 62 • **% Female:** NR • **Mean age:** 37 months • **Ethnicity:** C85%, B12%, A2%, H1% • **Mean maternal education:** NR • **Mean household income:** NR	• 5.0 (4.0)	• Intellectual Ability (NR) • Social Cognitive Ability (NR) • Social Competence Stranger (NR) • Social Competence Visitor (NR)	• **Statistics Extracted:** Partial Correlation • **Covariates:** age
Clarke-Stewart 2006[41]^Q^; Sample A: NICHD^Q^; Sample B: Korean	• **Sample A** • **Publication:** Journal (JADP) • **Design:** Longitudinal • **Dataset:** NICHD • **Country:** United States (National) • **Sample size:** class NR; child 119 • **% Female:** 53 • **Mean age:** 52–56 months • **Ethnicity:** C81%, B1%, A3%, H13%, O2% • **Mean maternal education:** 14.9 years • **Mean household income:** NR	• 8.08 (3.6)	• **Sample A** • ORCE-Self-Reliance 2.98 (0.64) • ORCE-Sustained Attention 2.95 (0.60) • CPSCS—Social Competence 28.90 (4.0) • TRF-Internalizing 6.88 (6.6) • TRF-Externalizing 8.3 (10.2)	• **Statistics Extracted:** Partial Correlation **Covariates:** maternal education, maternal employment
	• **Sample B** • **Publication:** Journal (AJDP) • **Design:** Cross-Sectional • **Country:** Korea • **Sample size:** class NR; child 90 • **% Female:** 49 • **Mean age:** 52–56 months • **Ethnicity:** A100% • **Mean maternal education:** 14.3 years • **Mean household income:** NR	• 14.5 (4.1)	• **Sample B** • ORCE-Self-Reliance 3.19(0.69) • ORCE-Sustained Attention 3.15 (0.65) • CPSCS—Social Competence 26.9 (4.4) • TRF-Internalizing 10.41 (9.5) • TRF-Externalizing 13.7 (13.3)	• **Statistics Extracted:** Partial Correlation • **Covariates:** maternal education, maternal employment
Colwell 2013[48]^N^	• **Publication**: Journal (ECRQ) • **Design**: Longitudinal • **Dataset:** Early Childhood Longitudinal Study-Birth Cohort (ECLS-B) • **Country**: United States • **Sample size:** class NR; child 1000 • **% Female:** 49 • **Mean age:** NR • **Ethnicity**: Hispanic 24%, Non-Hispanic Black 16% Non-Hispanic White 53%, Non-Hispanic Other 7% **Mean maternal education**: NR • **Mean household income**: NR	•.79 (3.26)	• ECLS Math -0.33 (.78) • ECLS Literacy -0.37 (0.73) • **Parent Report** • Social Competence 4.00 (0.55) • Emotional/Behavioral Reg. 2.42 (0.47) • Attention and Concentration 2.91 (0.40) • **Caregiver Report** • Social Competence 3.78 (0.58) Emotional/ Behavioral Reg. 1.88 (0.69) • Attention and Concentration 2.70 (0.50)	• **Statistics:** Beta • **Covariates:** child level—pretest score, gender, ethnicity, low birth weight, breastfed, well-child doctor visits, received WIC, lagged (a) health 9 mo. & 2 yr., (b) absence of common illness 9 mo. & 2 yr., (c) temperament 9 mo. & 2 yr., absence of injury; family level–maternal (a) birthplace not US, (b) other child ages < 6, (c) other children 6–18 years, (d) employment status, (e) marital status, (f) age, family received (a) TANF, (b) food stamps; community level–region, urbanicity; centre level–provider’s (a) gender, (b) age, (c) ethnicity, (d) experience, (e) ECE certificate, hours per week in care, months in care, center (a) type, (b) accreditation status, (c) licence status for what size, (d) center accepts subsidies (11)
Dotterer 2012[31]^m^^,^^A^	• **Publication:** Journal (ECDC) • **Design:** Longitudinal • **Dataset:** NCEDL (Multi-State & SWEEP) • **Country:** United States • **Sample size:** class 716; child 3584 • **% Female:** 51.17 • **Mean age:** 48 months • **Ethnicity:** C41%, B18%, H27%, O14% • **Mean maternal education:** 12.62 years • **Mean household income:** $36,041	• 7.57 (3.14)	• ARS Lang./Literacy 2.63 (0.87) • Naming Letters 6.31 (3.52) • Numbers 12.95 (9.23) • OWLS Exp. 89.53 (11.13) • PPVT-R 91.01 (13.47) • WJ Rhyming 2.59 (3.32) • WJ AP 95.64 (12.24)	• **Statistics Extracted:** B, SE • **Covariates:** child level—gender, ethnicity, maternal education; classroom level–hours per day, % Caucasian, poverty, program, poverty x program, teacher education, ECERS–Language & Interaction, ECERS–Provision for Learning, CLASS—Emotional Climate, CLASS–Instructional Climate
Downer 2012[32]: Whole Sample^A^: Sample A: Dual Language Learners^A^: Sample B: Latino^A^	• **Publication**: Journal (ECRQ) • **Design**: Longitudinal • **Dataset:** NCEDL (Multi-State & SWEEP) • **Country**: United States • **Sample size:** class 721 for NCEDL, current sample NR; Sample A child 956, Sample B child 328 • **% Female:** 51 • **Mean age:** NR • **Ethnicity:** C40%, B18%, H26%, O16% • **Mean maternal education**: 12.6 years • **Mean household income**: NR (over 58% of families were at or below 150% of the federal poverty threshold)	• 8.65 (3.54)	• Whole Sample • ARS Lang./Literacy 3.0 (0.97) • TCRS-SS 0.77 (3.64) • TCRS-PB 1.57 (0.75) • WJ-III-AP 412.19 (18.88) • Identifying Letters 12.9 (9.61)	• **Statistics:** B, SE, T-Test • **Covariates:** child/family level–pre-test score, gender, ethnicity, maternal education, poverty, language, test interval, test in Spanish, DLL status; classroom level—teacher education (BA), poverty, full-day, state, teacher/teacher’s assistant speaks Spanish, percent DLL, CLASS—Emotional Support, CLASS—Instructional Support, CLASS—Classroom Organization, CLASS by group
Dunn 1993[18]^S^	• **Publication:** Journal **(**ECRQ) • **Design:** Longitudinal • **Country:** United States **(**Central Indiana) • **Sample size:** class 30; child 60 • **% Female:** 57 • **Mean age:** 51.85 months • **Ethnicity:** C94%, B6% • **Mean maternal education:** 13.4 years • **Mean household income:** 29–34 K	• 12.82 (2.77)	• CBI Intellectual 53.88 (20.24) • CBI Sociability 33.87 (15.67) • PBQ Hyperactivity-Distractibility 13.78 (8.96) • PSI 44.8 (9.2)	• **Statistics Extracted:** B, Pearson’s Correlation, Partial Correlation • **Covariates:** age, marital status, income
Holloway 1988[42]^P^	• **Publication:** Journal (ECRQ) • **Design:** Longitudinal • **Dataset:** same as Holloway 1989 • **Country:** United States • **Sample size:** class 15; child 55 • **% Female:** 45.5 • **Mean age:** 53 months • **Ethnicity:** C94.5%, B3.6%, A1.8% • **Mean maternal education:** 15.9 years • **Mean household income:** NR	• 7.94 (1.8)	• SPSSP-Prosocial Responses 8.02 (6.05) • SPSSP-Prosocial Categories 1.98 (1.25) • SPSSP-Antisocial Responses 0.81 (1.87) • SPSSP-Antisocial Categories 0.33 (0.58)	• **Statistics Extracted:** Pearson’s Correlation • **Covariates:** none
Holloway 1989[43]^P^	• **Publication:** Journal (AJDP) • **Design:** Longitudinal • **Dataset:** same as Holloway 1988 • **Country:** United States • **Sample size:** class 15; child, range by analyses 52–55 • **% Female:** 45.5 • **Mean age:** 53 months • **Ethnicity:** C94.5%, B3.7%, A1.8% • **Mean maternal education:** 15.9 years • **Mean household income:** NR	• 7.44 (1.43)	• Social Competence (NR) • SPSSP-Prosocial Responses (NR)	• **Statistics Extracted:** Pearson’s Correlation, Beta • **Covariates:** none
Howes 1997[21]^D^	• **Publication:** Journal (MPQ) • **Design:** Cross-Sectional • **Dataset:** CQO • **Country:** United States (National) • **Sample size:** class 655; child 760 • **% Female:** 47 • **Mean age:** 51 months • **Ethnicity:** C65%, B15%, A4%, H6%, O14% • **Mean maternal education:** NR • **Mean household income:** NR	• NR	• CBI-Problem Behavior (NR) • PPVT-R (NR) • WJ-LWI (NR) • WJ-AP (NR)	• **Statistics Extracted:** F-Ratio • **Covariates:** maternal education, classroom
Howes 2008[33]^A^	• **Publication:** Journal (ECRQ) • **Design:** Longitudinal • **Dataset:** NCEDL (Multi-State & SWEEP) • **Country:** United States • **Sample size:** class 692; child range by analyses 1787–2044 • **% Female:** 51 • **Mean Age:** 48 months • **Ethnicity:** C42%, O58% • **Mean maternal education:** 12.8 years • **Mean household income:** 58% poverty	• 8.6 (NR)	• Identifying Letters (NR) • ARS Language/Literacy (NR) • OWLS-Oral Expression (NR) • PPVT-R (NR) • WJ-AP (NR) • SSBPS-Social Competence (NR) • SSBPS-Behavior Problems (NR)	• **Statistics Extracted:** Pearson’s Correlation, B, SE • **Covariates:** child/family level—state, gender, child age at fall assessment, ethnicity, maternal education, poverty, number of people in the household; classroom level—teacher education (BA), in/out school, full/part-day, T-C relationship, quality composite
Love 1992[19]	• **Publication:** Report • **Design:** Longitudinal • **Dataset:** CSRS • **Country:** United States • **Sample size:** class 112; child 2649 • **% Female:** 50 • **Mean age:** 48 months • **Ethnicity:** C18.5%, B36.7%, A12.6%, H32%, O0.2% • **Mean maternal education:** NR • **Mean household income:** NR	• NR	• BPI (NR) • Antisocial (NR) • Depressed (NR) • Attention-Deficit (NR) • Immature/Dependent (NR)	• **Statistics Extracted:** F-Ratio • **Covariates:** pretest scores
Mashburn, Pianta 2008[20]^A^	• **Publication:** Journal (DP) • **Design:** Longitudinal • **Dataset:** NCEDL (Multi-State & SWEEP) • **Country:** United States • **Sample size:** Class 671; child range by analyses 2307 & 2439 • **% Female:** 51% female • **Mean age:** 48 months • **Ethnicity:** C46%, B21%, H17%, O15% • **Mean maternal education:** 12.9 years • **Mean household income:** 47% poor	• 87% met 10:1	• Letter Naming 13.9 (9.42) • OWLS-Oral Exp. 93.6 (13) • PPVT-III 96.3 (14.3) • WJ-Rhyming 3.65 (4.02) • WJ-AP 99.1 (12.9) • TCRS-Social Competence • 3.66 (0.77) • TCRS-Problem Behaviors • 1.49 (0.54)	• **Statistics Extracted:** B, SE • **Covariates:** child/family level—pretest scores, gender, ethnicity, mother’s education, poverty; classroom level–state, teacher (a) BA, (b) ECE/CD, (c) teacher’s aide has CDA degree, group size is 20, comprehensive curriculum; program level—serves meals, has health services, provides family services
Mashburn 2009[34]^A^	• **Publication:** Journal (CD) • **Design:** Longitudinal • **Dataset:** NCEDL (Multi-State & SWEEP) • **Country:** United States • **Sample size:** class 453; child range by analyses 1680 & 1681 • **% Female:** 51% female • **Mean age:** 48 months • **Ethnicity:** C52%, B23%, H11%, O15% • **Mean maternal education:** 13.1 years • **Mean household income:** NR	• 7.67 (3.32)	• OWLS-Oral Exp. 94.9 (12.7) • PPVT-III 97.9 (14.1)	• **Statistics Extracted:** B, SE • **Covariates:** pretest score, gender, ethnicity, maternal education, peer language, full-day program, group size, CLASS—Emotional Support
McCartney 1984[44]^C^	• **Publication:** Journal (DP) • **Design:** Cross-Sectional • **Dataset:** Same as Phillips 1987 • **Country:** Bermuda • **Sample size:** class NR; child range by analyses 46–131 • **% Female:** NR • **Mean age:** 36–68 months • **Ethnicity:** C:20%, B:80% • **Mean maternal education:** 12.2 years • **Mean household income:** NR	• 10.5 (NR)	• ALI 3.1 (0.7) • Communication Task 34.4 (12.9) • PPVT-R 82.8 (16.7) • PLAI 1.3 (0.5)	• **Statistics Extracted:** Pearson’s Correlation • **Covariates:** none
NICHD ECCRN 1999[15]^Q^	• **Publication:** Journal (JAPH) • **Design:** Longitudinal • **Dataset:** NICHD • **Country:** United States (National) • **Sample size:** class NR; child range by analyses 110–250 • **% Female:** NR • **Mean age:** 36 months • **Ethnicity:** NR • **Mean maternal education:** NR • **Mean household income:** NR	• 6.98 (2.32)	• Bracken School Readiness (NR) • Reynell Scales Expressive (NR) • Reynell Comprehension (NR) • Behavior Problems Composite (NR) • Positive Social Behavior Composite (NR)	• **Statistics Extracted:** Adjusted Means, SE, F-Ratio • **Covariates:** ratio of income to needs, maternal sensitivity
Owen 2008[49]	• **Publication:** Journal (EED) • **Design:** Cross-Sectional • **Country:** United States • **Sample size:** class NR; child 223 • **% Female:** 48 • **Mean age:** 36–60 months • **Ethnicity:** B45%, H55% • **Mean maternal education:** 11.5 years • **Mean household income:** $19,157	• NR	• Bracken School Readiness (NR) • PPVT-III, TVIP (NR) • ASBI-Express (NR) • ASBI-Comply (NR) • CBCL-Internalizing (NR) • CBCL-Externalizing (NR) • STRS (NR) • TRF-Internalizing (NR) • TRF-Externalizing (NR)	• **Statistics Extracted:** Pearson’s Correlation • **Covariates:** none
Phillips 1987[45]^C^	• **Publication:** Journal (DP) • **Design:** Cross-Sectional • **Dataset:** same as McCartney 1984 • **Country:** Bermuda • **Sample size:** class NR; child, range by analyses 153–156 • **% Female:** NR • **Mean age:** NR (36–68 months) • **Ethnicity:** C:20%; B:80% • **Mean maternal education:** 12.2 years • **Mean household income:** NR	• 10.5 (NR)	• CBI Intelligence (NR) • CBI Considerateness (NR) • CBI Sociability (NR) • CBI Task Orientation (NR) • CBI Dependence (NR) • PBQ Aggression (NR) • PBQ Anxiety (NR) • PBQ Hyperactivity (NR)	• **Statistics Extracted:** R-Squared Change • **Covariates:** age at testing, values conformity, values social skills, age at entry, time in group care
Reid 2013[36]^A^	• **Publication**: Journal (EED) • **Design:** Longitudinal • **Dataset:** NCEDL (Multi-State & SWEEP) • **Country**: United States • **Sample size:** class 704; child 2,966 • **% Female:** NR • **Mean age:** NR • **Ethnicity:** NR • **2014 Mean maternal education**: 12.8 • **Mean household income**: 32, 574	• % classes with <10:1 • Low SES 86.79 • Mod SES 83.77 • High SES 89.08	• PPVT • Oral Expression Scale • WJ-III AP • TCRS—Social Competence	• **Statistics:** Beta • **Covariates:** child/family level–pretest score, gender, age, SES, ethnicity, single parent, ELL status, IEP status; classroom level–SES, deviation of income, % Caucasian, Instructional Quality, teacher has BA, teacher has more than a BA, class size (less than 18), full-day, Head Start, interaction terms (dropped due to insignificance–family income, mother’s education, % poor, days absent from preschool, assessment interval, ratios, teacher (a) had no BA (less than 4 year certificate), (b) CDA, (c) spoke Spanish; program offered (a) meals, (b) family services, (c) health services, (d) curriculum High Scope or Creative, (e) was located in public school, CLASS Emotional Support, ECERS)
Sabol 2013[35]^A^	• **Publication**: Journal (EED) • **Design**: Longitudinal • **Dataset:** NCEDL (Multi-State & SWEEP) • **Country**: United States • **Sample size:** class 673, child 2419 • **% Female:** • **Mean age:** 4.61 • **Ethnicity:** C42%, B25%, H18%, O15% • **Mean maternal education**: 12.96 years • **Mean household income**: NR	• 8.70 (3.10)	• WJ-Rhyming 3.36 (3.82) • Letter Knowledge 14.40 (9.34) • WJ-III AP 98.88 (13.37) • PPVT- III 95.52 (14.70) • OWLS-Expressive Lang. 93.21 (13.45) • Social Skills 3.56 (0.77) • Problem Behaviors 1.49 (0.55)	• **Statistics:** Pearson’s Correlation, Beta • **Covariates:** child/family level—pretest score, gender, ethnicity, maternal education, poverty, household size, attend pre-k prior year; classroom level -state, ethnicity, Head Start
Seppanen 1993[50]	• **Publication:** Report • **Design:** Longitudinal • **Dataset:** OSECP • **Country:** United States • **Sample size:** class 55; child 673 • **% Female:** 52 • **Mean age:** NR 48 months • **Ethnicity:** C15%, B48%, H31%, O7% • **Mean maternal ed.:** 66% high school or less • **Mean household income:** 78% children eligible for free or reduced price lunch	• 9.3 (2.7)	• PSI (NR) • CBRS (NR)	• **Statistics Extracted:** Partial Correlation • **Covariates:** pretest scores
Studer 1992[51]	• **Publication:** Journal (SSCD) • **Design:** Cross-Sectional • **Dataset:** NLSY • **Country:** United States (National) • **Sample size:** class NR; child 89 • **% Female:** NR • **Mean age:** 36 and 48 months • **Ethnicity:** NR (Black, Hispanic, Caucasian) • **Mean maternal education:** NR • **Mean household income:** NR (high representation poor)	• 7.06 (2.88)	• PPVT-R (NR)	• **Statistics Extracted:** F-Ratio, Eta Squared • **Covariates:** none
Travers 1980[52]	• **Publication:** Report • **Design:** Longitudinal • **Dataset:** NDSC • **Country:** United States **(**urban areas sample) • **Sample size:** class 117; child 1383 (analyses at center level n = 54–57) • **% Female:** NR • **Mean age:** 36 and 48 months • **Ethnicity:** C30%, B65%, O5% • **Mean maternal ed.:** 59% high school or less • **Mean household income:** 50% of families with income under $6,000	• 6.8 (2.7)	• PPVT (NR) • PSI (NR)	• **Statistics Extracted:** Pearson Correlation, F-Ratio • **Covariates:** NR
Zellman 2008[53]^m^^,^^Z^	• **Publication:** Report • **Design:** Longitudinal • **Dataset:** Colorado QRIS • **Country:** United States () • **Sample size:** class 156; child 1368 • **% Female:** 50 • **Mean age:** 47.3 months • **Ethnicity:** 42% minority • **Mean maternal education:** 16% BA • **Mean household income:** $45,400	• 6.21 (1.98)	• PPVT-III 92.76 (14.89) • WJ-LWI 104.76 (16.728) • WJ-PC 115.71 (13.322) • WJ-AP 97.42 (14.392) • CBI Verbal 3.51 (0.879) • CBI Dependence 2.45 (0.806) • CBI Independence 3.79 (0.682) • CBI Considerateness 3.5 (0.87) CBI CBI Apathy 2.13 (0.733)CBI Task Orientation 3.4 (0.87) • CBI Creativity 3.74 (0.773) • CBI Distractibility 2.58 (0.87) • CBI Hostility 2.50 (1.118)	• **Statistics Extracted:** B, SE • **Covariates:** child/family level—age at assessment, hours in care, duration of care, learning problems, gender, family income, parent has B.A., minority status, family language not English, parents' child-rearing style; program level—Head Start, nonprofit, and level of intervention intensity as determined by Qualistar
Zill 2003[39]^K^	• **Publication:** Report • **Design:** Longitudinal • **Dataset:** FACES 2000 (Head Start) • **Country:** United States • **Sample size:** class 278; child range by analyses 957–2138 • **% Female:** NR • **Mean age:** NR • **Ethnicity:** NR • **Mean maternal education:** NR • **Mean household income:** At-risk sample	• 5.4 (NR)	• PPVT-III 89.1 (NR) • WJ-LWI 92.9 • Cooperative Behavior 16.58 (4.63) • BPI-Hyperactive 1.21 (1.47)	• **Statistics Extracted:** B • **Covariates:** child/family-level—age, sex, ethnicity, language, disability, mother-father family, neither birth parent in home, parent literacy, parent education, family income, welfare status, books in home, frequency of reading to child (13); classroom level—full-day class, AP individualizing score, ECERS-R Language, CIS, teacher (a) BA or AA, (b) experience, (c) DAP beliefs score, (d) ethnicity, (e) salary, parent education, family income, proportion non-minority, proportion language minority (13); program level—High Scope curriculum, creative curriculum, teacher salary, proportion non-minority children, parent education, family income, proportion language-minority children (7)
Zill 2006[40]^K^	• **Publication:** Report • **Design:** Longitudinal • **Dataset:** FACES 2000 (Head Start) • **Country:** United States • **Sample size:** class 278; child range by analyses 674–1729 • **% Female:** 50 • **Mean Age:** 36 and 60 months • **Ethnicity:** C35%, B32%, A1%, H28%, Mixed3%, O1% • **Mean maternal ed.:** 64% high school or less • **Mean household income:** 80% > 24 K	• 5.4 (NR)	• One to One Counting (NR) • Color Naming (NR) • Design Coping (NR) • PPVT-III (NR) • Story Print Concepts-Book Knowledge (NR) • WJ-LWI (NR) • WJ-AP (NR) • WJ-Dictation (NR) • SSRS-Social Skills 18.12 (4.28) • FACES-Social Awareness NR • FACES-Aggressive 1.49 (1.93) • FACES-Hyperactive 0.97 (1.40) • FACES-Withdrawn 2.05 (2.40)	• **Statistics Extracted:** B • **Covariates:** child/family level—age, gender, ethnicity, disability, parent education, family income, welfare status, language-minority family, mother-father family, neither birth parent in home, parent literacy, books in home, frequency of reading to child, one-year head start graduate; classroom level—ECERS-R Language, education, experience, teacher ethnicity, teacher salary, teacher beliefs, CIS, parent education, family income level, proportion language-minority, proportion non-minority, full-day class; program level—parent education, family income, high/scope curriculum, creative curriculum, teacher salary, proportion non-minority children

Abbreviations: NR = Not Reported; C = Caucasian, B = African American, H = Hispanic, A = Asian, M = Mixed, O = Other. For all other acronyms, please refer to Supplemental Information 4 (S4 File) for all child outcomes, and S5 File for all journal, large study, or covariate acronyms.

^a^ Descriptives provided reflect characteristics (actual or estimates) of the sample/research design for which data was extracted for the current study and therefore may represent a subsample/analysis of the larger study.

^b ^This paper is one of a series of Meta-Analyses and Systematic Reviews assessing the relationship between child care quality and children’s outcomes; therefore, superscript letters below are in reference to various large databases that samples in these papers were drawn from. These letters have been kept consistent across the series for our readers.

^c ^Child-staff ratio values indicate the number of children per one staff member. Therefore, a higher number indicates that there are more children with fewer adults within a classroom, suggesting lower ECEC quality.

^d ^Scale of measurement for the means and standard reported in this table varied across studies (e.g., percentiles, standard scores, raw score). All outcomes used in the current paper are presented in S4 File.

^e ^All covariates used in the described sample are listed, but may vary by analyses.

^m ^Studies included in the meta analyses.

^A ^National Center for Early Development and Learning Dataset (NCEDL, 2002, 2004);

^C ^Bermuda Preschool Study (1980);

^D ^Cost, Quality and Outcomes Study (CQO, 1993–1994);

^K ^Head Start Family and children Experiences Survey (FACES, 2000) Cohort;

^**N**^ Early Childhood Longitudinal Study (ECLS-B, 2001–2006, Birth Cohort);

^P ^Northeastern United States sample (Holloway and colleagues, 2008; Year NR);

^Q ^National Institute of child Health and Human Development (NICHD, 1995–1996;

^S ^8-County Region of North-Central Indiana (Year NR);

^T ^Otitis Media Study (Year NR);

^Z ^Colorado QRIS

Across studies the average child-staff ratios ranged from 5 [6] to 14.5 [41] preschool-aged children per adult with a mean of 8.65. The average standard deviations across the studies ranged from 1.43 to 4.1 but were only reported in 20 of the 31 samples (see Table 3 for more information). To assess the variability of ratios within studies, we computed a difference score, *M* = 8.35, and median = 13 (calculated using the range for that study as the maximum score minus the minimum score). Range scores for ratios was reported in only 13 of the 31 samples. The lowest variance (difference score of 2.2) was for ratios ranging from 4.2 to 6.4 preschool-aged children per adult [52], and the highest variance (difference score of 33) was for ratios ranging from 1 to 34 preschool-aged children per adult [41]. These finding suggest substantial variance within and across studies, with some classrooms having ratios well above the standard guidelines (see Table 1).

Four studies [41,44,45,47] included ECEC programs located outside of the United States. Five studies [21,44,45,49,51] used a cross-sectional design. Twenty-four studies used a longitudinal design [14,15,17–20,31–43,47,48,50,52,53]. Of the independent samples, (i.e., excluding overlapping datasets by retaining only the largest sample size), the total sample size was 15,191 preschool-aged children (range 51 to 3584, median = 532).

### Measurement of Child-Staff Ratios

The way child-staff ratios were assessed varied between studies. In 15 of the studies [14,15,19,21,31,36,39–43,49,50,52] child-staff ratios were collected by research assistants who counted (often multiple times, at set intervals) the number of children and adults present in each classroom during classroom observations. Other studies relied on either staff [17,20,32,34,35,38,44,45,47,48] or maternal [51] reports. In one study, information on ratios was collected through both observations and staff reports and the highest number obtained for each classroom was utilized in the analyses [18]. In another study, ratios were initially collected via staff reports; however, after concerns surfaced about reliance of self-reported ratio counts, a sign-in/sign-out procedure for staff in the remaining classrooms was implemented [53]. One study did not present information as to how child-staff ratios were obtained [33]. Some studies included all adults present in a given classroom in their calculations of child-staff ratios regardless of whether the adult was an aide, volunteer, or parent [15,39,40]. Others used ambiguous terms such as counting “caregivers” [52] or “staff” [42,43], while others only included staff who were “actively interacting with children” [39,40] and one study [15] limited calculations to “all adults who regularly worked more than 10 hours per week”. However, most studies did not provide details about which adults were included in the ratio counts.

Seven studies [14,15,19–21,36,37] used a dichotomous child-staff ratio variable in their analyses to indicate whether or not a specific standard for ratios had been met. Point-biserial correlation coefficients were utilized for analyzing dichotomous variables. This type of correlation is mathematically equivalent to the Pearson (product moment) correlation that is used for continuous and ordinal variables. Thus, studies using either absolute values or binary variables were included within a given meta-analysis. Finally, for all samples used in this analysis, we ensured that lower child-staff ratios indicate less children per staff member (i.e., more favorable conditions). For those studies in which the original data reflected the opposite (i.e., a higher ratio represented less children per staff) [15,18,19,21,31,32,36–38,45,51–53], the direction of the statistical associations were adjusted following data extraction to ensure consistency across the reporting of all results.

### Systematic Review

**Fig 2:**
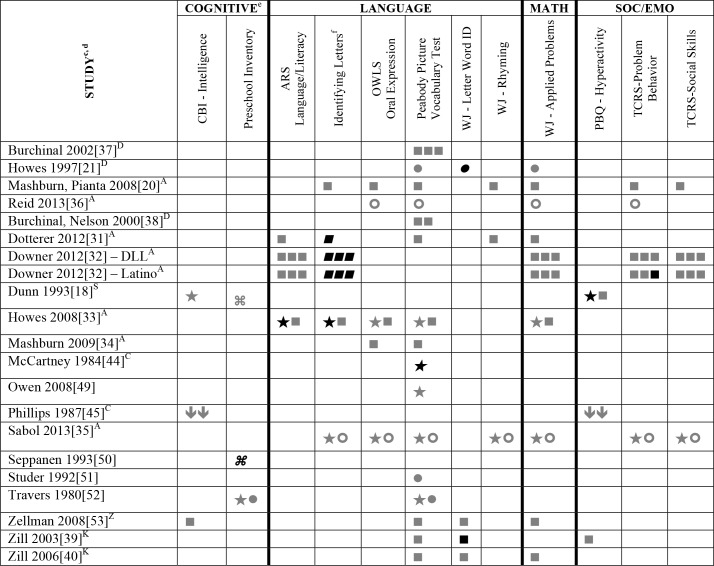
Systematic review results for the associations between child-staff ratios and child outcomes in 3 or more samples. ^a^ Abbreviations: Symbols bolded are significant and positive, symbols bolded and italicized are significant and negative, and symbols in grey are non-significant. Star = Zero Order Pearson’s Correlation, Unfilled circle = Beta, Filled square = Unstandardized Coefficient, Black diamond minus white X = T-Test, Key clover = Partial Correlation, Downward arrow = Effect Size, Filled circle = F-Ratio. ^**a**^To improve the readability of this complex table, 9 papers [15,17,19,37,41–43,48] that had an outcome that appeared in only that one paper were omitted from this table. Several analyses from other papers that had idiosyncratic outcomes are also excluded. For a comprehensive display of all of the data for all of the child outcomes see Tables A-D in S3 File. ^**b**^Ratio scores have been adjusted to be consistent across all data. In keeping with how ratios were operationalized in most of the papers we reviewed, we reverse scored when necessary so that lower ratio scores indicated fewer children per adult across all studies. Thus, negative relationships reflect an association between better ratios and better outcomes. In the case of problem behaviors, we expected a positive association as this reflects a correlation between better ratios and lower rates of problem behaviors [18,21,31,32,36–38,45,51,52].^**c**^This paper is one of a series of Meta-Analyses and Systematic Reviews assessing the relationship between child care quality and children’s outcomes; therefore, superscript letters below are in reference to various large databases that samples in these papers were drawn from. These letters have been kept consistent across the series for our readers. ^**d**^Samples within papers are described in more detail in Table 2. ^**e**^Acronyms for child outcomes are listed in S4 File. ^**f**^Identifying Letters (also refers to Identifying Letters, Naming Letters, and Letter-Naming Test). ^**A**^National Center for Early Development and Learning Dataset (NCEDL, 2002, 2004); ^**C**^Bermuda Preschool Study (1980); ^**D**^Cost, Quality and Outcomes Study (CQO, 1993–1994); ^**K**^Head Start Family and children Experiences Survey (FACES, 2000) Cohort; ^**S**^8-County Region of North-Central Indiana (Year NR); ^**Z**^Colorado QRIS.

All 29 eligible papers were systematically reviewed. The associations between child-staff ratios and child outcomes are reported using various statistical approaches and are reported in full in Tables A-D in S3 File. A snapshot of the results is also provided below in Fig 2, which displays the results obtained for only those outcomes that were used in three or more samples. In the 29 included studies, 209 distinct statistical analyses quantifying the association between child-staff ratios and measures of child outcomes were reported. These statistical analyses included 83 unique child outcome measures (S4 File). These were associated to ratios [17,33–35,39–44,47–50,53] operationalized in a variety of ways including number of staff divided by the number of children [18,31,32,38,45,52] the number of children divided by the number of staff or met/did not meet a specific standard (6:1, 7:1, 8:1 or 10:1) [14,15,20,21,36,37]. In addition, one study compared groups of classrooms with the highest (1:9.5) to lowest (1:7.2) ratio scores [19], and another compared groups of classrooms for ratios at different levels (1–6, 7–9, and 10–16) [51].

### Approach to learning outcomes

Two studies [45,53] reported associations between child-staff ratios and outcomes that look at children’s approach to learning (see Table A in S3 File). Zellman et al. [53] used five subscales of the Child Behavior Inventory measure and Phillips et al. [45] only used the Dependence and Task Orientation subscales from this measure. Both studies showed nonsignificant associations between ratios and this type of child outcome.

#### Cognitive outcomes

Ten studies reported associations between child-staff ratios and cognitive outcomes measured using 7 different instruments (see Table A in S3 File). Most of these measures were only used in a single study with the exception of the Intelligence subscale of the Child Behavior Inventory (*n* = 3) and Preschool Inventory (*n* = 3). The majority of these studies found no association between child-staff ratios and this type of child outcome. Only 2 studies reported significant relationships, with better child-staff ratios related to better cognitive child outcomes [14,50].

#### Physical outcomes

Zill et al. [40] reported on the relationship between child-staff ratios and a child physical development outcome. The outcome in this study was measured using the Design Copying instrument. The association between the variables of interest was not significant.

#### Combination outcomes

One study reported the relationship between child-staff ratios and the Child Behavior Rating Scale [50]. The results of this study were nonsignificant.

#### Math outcomes

Associations between child-staff ratios and 5 different math outcomes were reported in 12 studies (see Table A in S3 File). The WJ AP measure of child competency in mathematics is the only outcome that was reported in a large number of studies (*n* = 10), see Fig 2. The rest of the math outcomes were reported in only one study. Most of the results reported across these studies were nonsignificant suggesting a lack of relationship between child-staff ratios and math outcomes. Two studies reported significant results. However, while Colwell et al. [48] reported that higher child-staff ratios were related to better math outcomes, Anders et al. [47] reported that lower child-staff ratios were related to better math outcomes.

#### Language outcomes

The associations between child-staff ratios and child language outcomes were reported for 21 studies included in this systematic review (see Table B in S3 File). The results for 19 different measures of language development were reported. Most of these measures were used in a single study. Only 7 measures were used in 2 or more studies (see Fig 2). The PPVT was reported in the largest number of studies (*n* = 16).

The vast majority of the results in these studies showed nonsignificant associations between child-staff ratios and child language outcomes. Among the small number of significant results that were reported, some [14,21,31,32,44] showed that lower child-staff ratios were related to better child language outcomes, while others [33,39] showed that higher child-staff ratios related to better child language outcomes.

#### Social-emotional: positive behavior outcomes

Studies looking at the relationship between child-staff ratios and child positive behavior reported findings for 22 different outcomes in 18 studies (see Table C in S3 File). Most of these outcomes were only used in a single study. Most studies involved multiple indicators of positive behavior and child-staff ratios. Four outcomes (CBI–Considerateness and Sociability, SPSSP—Prosocial Responses, and Teacher Child Rating Scale–Social Competence) were reported in 2 and 4 studies respectively.

For most positive behavior outcomes reported in a single study the associations were nonsignificant. Of the significant results some [15,41,45,48] indicated that the classrooms with the smaller number of children per staff had children with more positive behavior outcomes (see Table C in S3 File).

#### Social-emotional: problem behavior outcomes

Problem behavior was measured in 16 studies with 23 different outcome variables (see Table D in S3 File). With the exception of the Behavior Problems subscale from the TCRS that was used in 4 studies, the TRF CBCL—Externalizing and Internalizing subscales that was found in 2 studies and the PBQ–Hyperactivity scale that was reported in 2 studies, all other outcomes were used in a single study.

The majority of the reported results showed no significant association between child-staff ratios and problem behavior outcomes. A few significant results revealed fewer child behavior problems (or less children with problem behavior) in classrooms with lower ratios [15,18,32,41]. In contrast, one study reported more children with behavior problems in classrooms with lower ratios [45].

Overall, the vast majority of the results reported in the 29 studies reviewed as part of this systematic review suggest small or no associations between child-staff ratios and children’s cognitive, language, and social-emotional outcomes. Our examination of study characteristics such as publication year, study design and operationalization of child-staff ratios showed no patterns of association across the studies. Significant associations were reported for statistics that do (e.g., beta coefficients from regression) and do not (e.g., Pearson correlations and F-ratios) control for covariates.

### Meta-Analyses

**Fig 3:**
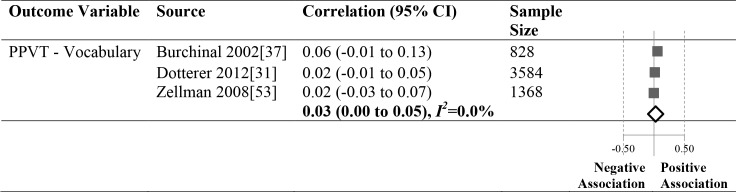
Meta-analysis results for the associates between child-staff ratios and child language outcome.

Eighty-three different child outcomes were reported in the included studies (see S4 File for a complete list of outcome measures across all 29 studies). Outcome measures varied substantially in terms of the skill/ability being assessed (e.g., inattention; receptive language; counting task), informant (e.g., child assessment, teacher report, parent report), and psychometric properties (e.g., standardized norm-referenced measures vs. tasks developed by authors). Of the 83 child outcome measures reported in our sample of papers only the PPVT was utilized in three or more studies with statistics that enabled us to conduct meta-analyses (see Fig 3 and S4 File).

The association between child-staff ratios and the PPVT was not significant (pooled correlation coefficient 0.03 95% CI: 0.00 to .05). While this meta-analysis is based on only three studies, together they represent 5780 participants. The lack of association was consistent across the included studies with a very low value for the index of heterogeneity (*I*^*2*^ = 0).

## Discussion

Researchers have been examining early childhood education and care settings for decades. Yet, we still lack an acceptable empirically-based directive for stakeholders regarding the effects of quality of care on children’s development [54]. Conclusions concerning which aspects of quality matter most for children are often deduced from a few select intensive early intervention projects conducted in the 1960’s (i.e., The High Scope Perry Preschool Project) and 1970’s (i.e., The Carolina Abecedarian Project). The populations served and scope of services provided in these studies call into question their generalizability and applicability to today’s ECEC programs. In the several decades that have passed since these seminal longitudinal studies were undertaken, extensive research has documented mixed results. We conducted very comprehensive searches that lead to a review of 29 studies. This systematic analysis revealed that within the range of ratios found in the literature, variations in child-staff ratios for pre-school aged children classrooms have small, if any, associations with concurrent or subsequent child outcomes. Ratios showed substantial variability both within the studies that we included and across them. Therefore, the lack of associations between ratios and child outcomes found in our review are not explained by limited ranges of the ratios observed in the samples. Based on the small number of studies available for meta-analysis, no association was found between child-staff ratios and children’s receptive language as measured by the PPVT. However, it is important to note that the studies included in this review only included ratios that met local regulations. Thus, findings from this analysis cannot be used to argue for relaxing existing child-staff ratio regulations.

To our knowledge this is the first attempt to systematically review and meta-analyze this highly complex and heterogeneous literature. Strengths of our review include a very comprehensive search strategy, strict inclusion and exclusion criteria to screen the appropriate literature with a primary aim to systematically review and only meta-analyze homogeneous studies. However, this review also suffers from a number of limitations that mainly stem from the methodological issues of the primary research covered in this review.

A number of conceptual explanations that may contribute to the lack of associations between child-staff ratios and children’s outcomes are described below. The association in question may be curvilinear [55] or “J” shaped whereby thresholds must be met for quality to impact children’s outcomes. None of the studies we reviewed empirically explored this question. However, a systematic comparison of the results from those studies whose mean child-staff ratios fell within the lowest, highest, and mid-range of values in our systematic review sample did not produce evidence to support a curvilinear trend between ratios and child outcomes (results not shown).

It is possible that some children are more affected than others by environmental circumstances, a concept referred to as *differential susceptibility* [56–58]. Thus, an interaction may exist between child characteristics (e.g., temperaments, family demographics) and the impact of ratios on child outcomes. Unfortunately, data were not available to enable us to assess this hypothesis. However, our qualitative synthesis did not suggest that findings systematically varied by the composition of the sample (i.e., children from at-risk backgrounds), publication year, or study design (i.e., longitudinal vs. cross-sectional). Nonetheless, researchers are encouraged to utilize study designs and statistical analyses that allow for the investigation of moderation, mediation, and ecological transactional models [59] in order for the ECEC literature to go beyond investigating the effect of ratios on the ‘average’ child.

Future research is needed to determine whether teacher characteristics (e.g., level of education; years of experience) and classroom/program variables moderate the effect of ratios on child development.

Finally, there is a mismatch in the unit of measurement in this literature as predictors (i.e., quality indicators) are almost always measured at the classroom level while child outcome variables are examined at the individual level [60]]. This may introduce imprecision in the linkages between quality and child outcomes. Advanced statistical techniques can account for different levels of measurement. However, the conceptual issue as to whether a classroom aggregate measure that estimates the average child’s experience of quality would be expected to impact the outcomes of an individual child remains.

As noted above, this area of research suffers from a number of methodological limitations. These methodological limitations within the ECEC literature may explain the lack of statistical associations between child-staff ratios and child outcome measures. In fact, due to these methodological issues and to limited variability in methodological quality between studies, a risk of bias assessment yielded non-informative results. As described below, methodological issues included: i) how child-staff ratios were operationalized; ii) the domains of child outcomes measured; iii) the psychometric properties of the outcome measures; and iv) study design.

There was enormous variability in the method of ratio data collection (e.g., via observations or staff/parent reports), the adults included in ratio counts, and how ratios were calculated. Previous research suggests that the validity of child-staff ratio measurements can be improved by lengthening the observation period [61] as ratios fluctuate over the course of the day [61,62]. Until consistent, empirically-based methods to measure ratios are adopted by ECEC researchers (and fully described in their papers), differences in ratios across studies may simply be an artifact of discrepancy in measurement approaches rather than actual variations in the numbers of children and adults present in each room.

Eighty-three different child outcome measures were identified in the studies we reviewed. Despite the large number of outcomes found in this literature, many key domains of child development are under-represented. This is consistent with a review of sixty-five studies of ECEC quality published between 1979 and 2005 [60], which like the current study, found an emphasis on socioemotional, language and cognitive development with limited consideration of motivational aspects of learning (“approaches to learning” such as task persistence or enthusiasm), physical development, or health outcomes. We argue that future research on the impact of ECEC quality on outcomes should focus on established measures with good psychometric properties to assess specific aspects of child development that are conceptually linked to the specific aspect of ECEC quality in question. Such an approach will go a long way towards helping researchers synthesize information across research in this area in the future.

The studies included in this review exhibited many measurement problems. Simply reporting information related to the reliability and validity of the child outcome measure(s) would be an important first step in addressing the methodological problems present in this area of research [60].

Finally, all but one of these studies used an observational design and therefore causal conclusions regarding the effect of ratios on child outcomes cannot be made. The one exception, the California Staff/Child Ratio Study [19] involved random assignment of children into groups with different ratio levels. Staff reports of antisocial, depressed, attention-deficit, and immature/dependent behaviors for each child in the classroom were not linked to better/worse ratios after controlling for baseline behavior scores. However, the period in which classrooms operated at the different ratios levels was relatively short in this study. It is imperative that researchers design studies that are more methodologically rigorous in the future. Conducting research on this highly regulated quality indicator is not easy and may require partnerships with policy makers and regulatory agencies involved in the oversight of ECEC programs.

## Conclusion

Despite the substantial limitations of research in this area, the current study suggests that, within the range of permissible child-staff ratios, variations in ratios have small, if any, associations with concurrent and subsequent child outcomes. The small number of significant associations between child-staff ratios in preschool-aged classrooms and children’s developmental outcomes that were reported may reflect selection biases. Specifically, they may reflect family-level factors that play a role in child care selection, such as maternal education or family income, rather than true child care effects. However, as noted above, the research available for this systematic review included studies with significant methodological limitations and only studies with child-staff ratios that fell within current regulations. Thus, our findings should not be interpreted as indicating that regulation of ratios can be relaxed in any way. Rather, we emphasize that within the range of what is currently permissible by licensing regulations, better ratios in preschool-aged ECEC classrooms are not associated with better outcomes for children. This is consistent with findings from large-scale meta-analyses examining teacher-student ratios and student achievement in formal education systems [63]. While we stress that these findings should not be overstated, they do suggest that other areas of investment in quality improvement in ECEC programs, such as staff professional development, may yield better payoffs for the many stakeholders impacted by ECEC quality. In addition, our results indicate a strong need for comparative effectiveness type of research designs on this issue in multiple settings. These include prospective cohorts or cluster randomized studies, with different ratios and prospective criterion-specific evaluations of consumer (parents)-centric outcomes to guide future practice in this area, which affects wide-scale populations.

## Supporting Information

S1 File **Search Syntax RATIO, Tables A-D**.
(PDF)

S2 File Formulas RATIO. (PDF)

S3 File **Systematic Review RATIO, Tables A-D**.
(PDF)

S4 File Child Outcomes RATIO. (PDF)

S5 File Acronyms RATIO. (PDF)

S6 File PRISMA Checklist RATIO. (PDF)

S7 File Database RATIO. (ZIP)
