# Supplementary material for: Child-Staff Ratios in Early Childhood Education and Care Settings and Child Outcomes: A Systematic Review and Meta-Analysis
Source: PLoS One. 2017 Jan 19;12(1):e0170256. doi: 10.1371/journal.pone.0170256 (PMC5245988; doi:10.1371/journal.pone.0170256)
Supplement: S2 File — (PDF) [file pone.0170256.s002.pdf]

Child-Staff Ratios in Early Childhood Education and Care Settings and Child Outcomes: A Systematic Review and Meta-Analysis

Supplemental Information 2

Formulas for Converting Statistics to r for Meta Analyses

Conversion to *t*, *d* or *r* through *t*-statistic

$$t = \frac{B}{SE(B)}$$

$$d = \frac{2t}{\sqrt{n-1}}$$

$$r = \frac{d}{\sqrt{d^2+4}}$$

where *B* is an unstandardized regression coefficient, *SE(B)* is its standard error, *d* is its standardized mean difference, *r* is the correlation effect size, and *n* is sample size.
