# Supplementary material for: Child-Staff Ratios in Early Childhood Education and Care Settings and Child Outcomes: A Systematic Review and Meta-Analysis
Source: PLoS One. 2017 Jan 19;12(1):e0170256. doi: 10.1371/journal.pone.0170256 (PMC5245988; doi:10.1371/journal.pone.0170256)
Supplement: S3 File — Systematic Review RATIO, Tables A-D. (PDF) [file pone.0170256.s003.pdf]

Supplemental Information 3
Systematic Review Results: All Outcomes

Table A. Child/Staff Ratio Predicting all Approach, Cognitive, Combo, Physical and Math Outcomes[a]

**STUDY**[b, c]	**APPROACH**[d]					**COGNITIVE**							**Combo**	**Physical**	**MATH**				
	CBI- Creativity	CBI- Dependence	CBI- Distractibility	CBI- Independence	CBI – Task Orientation	Bayley-Revised - Mental Development Index	Bracken Basic Concept Scale - School Readiness	CBI- Intelligence	FACES - Social Awareness	Identifying Colors	Intellectual Ability	Preschool Inventory	Child Behavior Rating Scale	Design Copying	ECLS - Math	K-ABC Arithmetic	Naming Numbers	One-One Counting	WJ - Applied Problems
Anders 2012[6]																■■■■■<br>■▰▰▰▰			
Burchinal, Roberts 2000[1][T]						★													
Clarke-Stewart 1994[7]											⌘								
Colwell 2013[8][N]															○				
Dotterer 2012[9][A]																	■		■
Downer 2012[10] – DLL[A]																			■■■
Downer 2012[10] – Latino[A]																			■■■
Dunn 1993[11][S]								★				⌘							
Howes 1997[2][D]																			●
Howes 2008[12][A]																			★■
Mashburn, Pianta 2008[3][A]																			■
NICHD 1999[4][Q]							☒												
Owen 2008[13]							★												
Phillips 1987[14][C]		⬇⬇			⬇⬇			⬇⬇											

Reid 2013[5][A]																			○
Sabol 2013[15][A]																			★○
Seppanen 1993[16]												⌘	⌘						
Travers 1980[17]												★●							
Zellman 2008[18][Z]	■	■	■	■	■			■											■
Zill 2006[19][K]									■	■				■				■	■

Legend for Table

Significant and Positive	Significant and Negative	Nonsignificant	Statistic
★	★	★	r - Zero Order Pearson's Correlation
■	■	■	B (Unstandardized Coefficient)
○	○	○	Beta
⌘	⌘	⌘	Partial Correlation
●	●	●	F-Ratio
⊠	⊠	⊠	Adjusted Means
⬇	⬇	⬇	Effect Size

[a]Ratio scores have been adjusted to be consistent across all data. In keeping with how ratios were operationalized in most of the papers we reviewed, we reverse scored when necessary so that lower ratio scores indicated fewer children per adult across all studies. Thus, negative relationships reflect an association between better ratios and better outcomes. In the case of problem behaviors, we expected a positive association as this reflects a correlation between better ratios and lower rates of problem behaviors [2,4,5,14,17].
[b]This paper is one of a series of Meta-Analyses and Systematic Reviews assessing the relationship between child care quality and children's outcomes; therefore, superscript letters below are in reference to various large databases that samples in these papers were drawn from. These letters have been kept consistent across the series for our readers.
[c]Samples within papers are described in more detail in the manuscript in Table 3.
[d]Acronyms for child outcomes are listed in the Supplemental Information 4 document.
[A]National Center for Early Development and Learning Dataset (NCEDL, 2002, 2004); [C]Bermuda Preschool Study (1980); [D]Cost, Quality and Outcomes Study (CQO, 1993-1994); [K]Head Start Family and children Experiences Survey (FACES, 2000) Cohort; [N]Early Childhood Longitudinal Study (ECLS-B, 2001-2006, Birth Cohort); [Q]National Institute of Child Health and Human Development (NICHD, 1995-1996); [S]8-County Region of North-Central Indiana (Year NR); [T]Otitis Media Study (Year NR); [Z]Colorado QRIS.

Supplemental Information 3
Systematic Review Results: All Outcomes

Table B. Child/Staff Ratio Predicting Language Outcomes[a]

STUDY[b, c]	Language[d]																		
	Adaptive Language Inventory	ARS – Language/Literacy	Book Knowledge	Communication Task	ECLS - Language/Literacy	Identifying Letters[e]	OWLS - Expressive Language	OWLS - Total Scale	Peabody Picture Vocabulary Test	Preschool Lang. Assessment	Reynell - Expressive Language	Reynell - Language Comprehension	SICD-Expressive Communication Age	SICD-Receptive Communication Age	Story & Print Concepts – Book Knowledge	WJ - Dictation	WJ - Letter Word ID	WJ - Passage Comprehension	WJ - Rhyming
Burchinal 2002[20][D]									■■■										
Burchinal, Nelson 2000[21][D]									■■										
Burchinal, Roberts 2000[1] [T]													★	☆					
Colwell 2013[8][N]					○														
Dotterer 2012[9][A]		■				▰		■	■										■
Downer 2012[10] – DLL[A]		■■■				▰▰▰													
Downer 2012[10] – Latino[A]		■■■				▰▰▰													
Howes 1997[2][D]									●								⬬		
Howes 2008[12][A]		★■				★■	☆■		☆■										
Mashburn, Pianta 2008[3][A]						■	■		■										■
Mashburn 2009[22][A]							■		■										
McCartney 1984[23][C]	☆			☆					★	☆									
NICHD 1999[4][Q]											☒	☒							
Owen 2008[13]									☆										

Reid 2013[5][A]							○ (grey)		○ (grey)										
Sabol 2013[15][A]						★○ (grey)	★○ (grey)		★○ (grey)										★○ (grey)
Studer 1992[24]									● (grey)										
Travers 1980[17]									★● (grey)										
Zellman 2008[18][Z]									■ (grey)								■ (grey)	■ (grey)	
Zill 2003[25][K]									■ (grey)								■		
Zill 2006[19][K]									■ (grey)						■ (grey)	■ (grey)	■ (grey)		

Legend for Table

Significant and Positive	Significant and Negative	Nonsignificant	Statistic
★	*★*	★ (grey)	r - Zero Order Pearson's Correlation
■	*■*	■ (grey)	B (Unstandardized Coefficient)
○	*○*	○ (grey)	Beta
●	*●*	● (grey)	F-Ratio
☒	*☒*	☒ (grey)	Adjusted Means

[a]Ratio scores have been adjusted to be consistent across all data. In keeping with how ratios were operationalized in most of the papers we reviewed, we reverse scored when necessary so that lower ratio scores indicated fewer children per adult across all studies. Thus, negative relationships reflect an association between better ratios and better outcomes. In the case of problem behaviors, we expected a positive association as this reflects a correlation between better ratios and lower rates of problem behaviors [2,4,5,9,10,17,20,21,24].

[b]This paper is one of a series of Meta-Analyses and Systematic Reviews assessing the relationship between child care quality and children's outcomes; therefore, superscript letters below are in reference to various large databases that samples in these papers were drawn from. These letters have been kept consistent across the series for our readers.

[c]Samples within papers are described in more detail in the manuscript in Table 3.

[d]Acronyms for child outcomes are listed in the Supplemental Information 4 document.

[e]Identifying Letters (also refers to Identifying Letters, Naming Letters, and Letter-Naming Test).

[A]National Center for Early Development and Learning Dataset (NCEDL, 2002, 2004); [C]Bermuda Preschool Study (1980); [D]Cost, Quality and Outcomes Study (CQO, 1993-1994); [K]Head Start Family and children Experiences Survey (FACES, 2000) Cohort; [N]Early Childhood Longitudinal Study (ECLS-B, 2001-2006, Birth Cohort); [Q]National Institute of Child Health and Human Development (NICHD, 1995-1996); [T]Otitis Media Study (Year NR); [Z]Colorado QRIS.

Supplemental Information 3
Systematic Review Results: All Outcomes

Table C. Child/Staff Ratio Predicting all Positive Behavior Outcomes[a]

**STUDY** [b, c]	**Positive Behavior**[d]																					
	ASBI - Comply	ASBI - Express	CBI - Considerateness	CBI - Sociability	Cooperative Behavior	CPSCS - Social Skills	ECLS - Attention & Concentration	ECLS – Emotional & Behavioral Regulation	ECLS - Prosocial	ORCE - Self-Reliance	ORCE- Sustained Attention	Positive Behavior (author created composite)	Social Cognitive Ability	Social Competence	Social Competence with Stranger	Social Competence with Visitor	SPSSP - Prosocial Categories	SPSSP - Prosocial Responses	SSBPS - Social Competence	SSRS - Social Skills	Student Teacher Relationship Scale	TCRS - Social Competence
Clarke-Stewart 1994[7]													⌘		⌘	⌘						
Clarke-Stewart 2006[26][Q]						***⌘***				⌘	⌘											
Colwell 2013[8][N]							○○	○ **○**	○ ○													
Downer 2012[10] – DLL[A]																						■■ ■
Downer 2012[10] – Latino[A]																						■■ ■
Dunn 1993[11][S]				★																		
Holloway 1988[27]																	★	★				
Holloway 1989[28]														★				★				
Howes 2008[12][A]																			★■			
Mashburn, Pianta 2008[3][A]																						■
NICHD 1999[4][Q]												☒										
Owen 2008[13]	★	★																			★	

	★	★																				
Phillips 1987[14][C]			⬇⬇	⬇⬇																		
Reid 2013[5][A]																						○
Sabol 2013[15][A]																						★■
Zellman 2008[18][Z]			■																			
Zill 2003[25][K]					■																	
Zill 2006[19][K]																				■■		

Legend for Table

Significant & Positive	Significant & Negative	Nonsignificant	Statistic
★	★	★	r - Zero Order Pearson's Correlation
■	■	■	B (Unstandardized Coefficient)
○	○	○	Beta
⌘	⌘	⌘	Partial Correlation
☒	☒	☒	Adjusted Means
⬇	⬇	⬇	Effect Size

[a]Ratio scores have been adjusted to be consistent across all data. In keeping with how ratios were operationalized in most of the papers we reviewed, we reverse scored when necessary so that lower ratio scores indicated fewer children per adult across all studies. Thus, negative relationships reflect an association between better ratios and better outcomes. In the case of problem behaviors, we expected a positive association as this reflects a correlation between better ratios and lower rates of problem behaviors [4,5,10,11,14].

[b]This paper is one of a series of Meta-Analyses and Systematic Reviews assessing the relationship between child care quality and children's outcomes; therefore, superscript letters below are in reference to various large databases that samples in these papers were drawn from. These letters have been kept consistent across the series for our readers.

[c]Samples within papers are described in more detail in the manuscript in Table 3.

[d]Acronyms for child outcomes are listed in the Supplemental Information 4 document.

[A]National Center for Early Development and Learning Dataset (NCEDL, 2002, 2004); [C]Bermuda Preschool Study (1980); [K]Head Start Family and children Experiences Survey (FACES, 2000) Cohort; [N]Early Childhood Longitudinal Study (ECLS-B, 2001-2006, Birth Cohort); [Q]National Institute of Child Health and Human Development (NICHD, 1995-1996); [S]8-County Region of North-Central Indiana (Year NR); [Z]Colorado QRIS.

Supplemental Information 3
Systematic Review Results: All Outcomes

Table D. Child/Staff Ratio Predicting all Problem Behavior Outcomes [a]

STUDY [b, c]	Problem Behavior [d]																						
	Behavior Problems (author created composite)	BPI - Antisocial	BPI - Attention-Deficit	BPI - Depressed	BPI - Immature/Dependent	CBCL - Externalizing	CBCL - Internalizing	CBI - Apathy	CBI - Behavior Problems	CBI - Hostility	FACES - Aggressive Behavior	FACES - Hyperactive Behavior	FACES - Withdrawn Behavior	PBQ - Aggressiveness	PBQ - Anxiety	PBQ – Hyperactivity	PBS - Hyperactive Behavior	SPSSP - Antisocial Categories	SSPSSP - Antisocial Responses	SSBPS - Behavior Problems	TCRS - Problem Behaviors	TRF CBCL - Externalizing	TRF CBCL - Internalizing
Clarke-Stewart 2006[26] [Q]																						⌘	⌘
Downer 2012[10] – DLL [A]																					■■<br>■		
Downer 2012[10] – Latino [A]																					■■<br>■		
Dunn 1993[11] [S]																★■							
Holloway 1988[27] [P]																		★	★				
Howes 1997[2] [D]									●														
Howes 2008[12] [A]																				★■			
Love 1992[29]		●	●	●	●																		
Mashburn, Pianta 2008[3] [A]																					■		
NICHD 1999[4] [Q]	☒																						
Owen 2008[13]						★	★															★	★
Phillips 1987[14] [C]														↓↓	↓↓	↓↓							
Sabol 2013[15] [A]																					★■		
Zellman 2008[18] [Z]								■		■													
Zill 2003[25] [K]																	■						
Zill 2006[19] [K]											■<br>■	■<br>■	■■										

Table D. Child/Staff Ratio Predicting all Problem Behavior Outcomes [a]

Legend for Table

Significant and Positive	Significant and Negative	Nonsignificant	Statistic
★	★	★	r - Zero Order Pearson's Correlation
■	■	■	B (Unstandardized Coefficient)
⌘	⌘	⌘	Partial Correlation
●	●	●	F-Ratio
⊠	⊠	⊠	Adjusted Means
⬇	⬇	⬇	Effect Size

[a]Ratio scores have been adjusted to be consistent across all data. In keeping with how ratios were operationalized in most of the papers we reviewed, we reverse scored when necessary so that lower ratio scores indicated fewer children per adult across all studies. Thus, negative relationships reflect an association between better ratios and better outcomes. In the case of problem behaviors, we expected a positive association as this reflects a correlation between better ratios and lower rates of problem behaviors [2,4,10,11,14,29].

[b]This paper is one of a series of Meta-Analyses and Systematic Reviews assessing the relationship between child care quality and children's outcomes; therefore, superscript letters below are in reference to various large databases that samples in these papers were drawn from. These letters have been kept consistent across the series for our readers.

[c]Samples within papers are described in more detail in the manuscript in Table 3.

[d]Acronyms for child outcomes are listed in the Supplemental Information 4 document.

[A]National Center for Early Development and Learning Dataset (NCEDL, 2002, 2004); [C]Bermuda Preschool Study (1980); [D]Cost, Quality and Outcomes Study (CQO, 1993-1994); [K]Head Start Family and children Experiences Survey (FACES, 2000) Cohort; [P]Northeastern United States sample (Holloway and colleagues, 2008; Year NR); [Q]National Institute of Child Health and Human Development (NICHD, 1995-1996); [S]8-County Region of North-Central Indiana (Year NR); [Z]Colorado QRI

References

1. Burchinal M, Roberts J, Riggins Jr R, Ziesel S, Neebe E, Bryant D. Relating quality of center based child care to early cognitive and language development longitudinally. Child Dev. 2000;71: 339-357. doi:10.1111/1467-8624.00149.

2. Howes C. Children's experiences in center-based child care as a function of teacher background and adult: child ratio. Merrill-Palmer Q. 1997;43: 404-425.

3. Mashburn A, Pianta R, Hamre B, Downer JT, Barbarin OA, Bryant D, et al. Measures of classroom quality in prekindergarten and children's development of academic, language, and social skills. Child Dev. 2008;79: 732-749. doi:10.1111/j.1467-8624.2008.01154.x.

4. NICHD Early Child Care Research Network. Child outcomes when child care center classes meet recommended standards for quality. Am J Public Health. 1999;89: 1072-1077. doi:10.2105/AJPH.89.7.1072.

5. Reid J, Ready D. High-quality preschool: the socioeconomic composition of preschool classrooms and children's learning. Early Educ Dev. 2013;24: 1082-1111. doi:10.1080/10409289.2012.757519.

6. Anders Y, Rossbach H, Weinert S, Ebert S, Kuger S, Lehrl S, et al. Home and preschool learning environments and their relations to the development of early numeracy skills. Early Child Res Q. 2012;27: 231-244. doi:10.1016/j.ecresq.2011.08.003.

7. Clarke-Stewart K, Gruber I, Fitzgerald L. Children at home and in day care. Hillsdale, NJ: Lawrence Erlbaum Associates, Inc; 1994.

8. Colwell N, Gordon R, Fujimoto K, Kaestner R, Korenman S. New evidence on the validity of the Arnett Caregiver Interaction Scale: results from the Early Childhood Longitudinal Study-Birth Cohort. Early Child Res Q. 2013;28: 218-233. doi:10.1016/j.ecresq.2012.12.004.

9. Dotterer A, Burchinal M, Bryant D, Early D, Pianta R. Universal and targeted pre-kindergarten programmes: a comparison of classroom characteristics and child outcomes. Early Child Dev Care. 2012;183: 931-950. doi:10.1080/03004430.2012.698388.

10. Downer J, López M, Grimm K, Hamagami A, Pianta R, Howes C. Observations of teacher–child interactions in classrooms serving latinos and dual language learners: applicability of the classroom assessment scoring system in diverse settings. Early Child Res Q. 2012;27: 21-32. doi:10.1016/j.ecresq.2011.07.005.

11. Dunn L. Proximal and distal features of day care quality and children's development. Early Child Res Q. 1993;8: 167-192. doi:10.1016/S0885-2006(05)80089-4.

12. Howes C, Burchinal M, Pianta R, Bryant D, Early D, Clifford R, et al. Ready to learn? children's pre-academic achievement in pre-kindergarten programs. Early Child Res Q. 2008;23: 27-50. doi:10.1016/j.ecresq.2007.05.002.

13. Owen M, Klausli J, Mata-Otero A, Caughy M. Relationship-focused child care practices: quality of care and child outcomes for children in poverty. Early Educ Dev. 2008;19: 302-329. doi:10.1080/10409280801964010.

14. Phillips D, McCartney K, Scarr S. Child-care quality and children's social development. Dev Psychol. 1987;23: 537-543. doi:10.1037/0012-1649.23.4.537.

15. Sabol T, Hong S, Pianta R, Burchinal M. Can rating pre-k programs predict children's learning? Science. 2013;341: 845-846. doi:10.1126/science.1233517.

16. Seppanen P, Godin K, Metzger J, Bronson M, Cichon D. Observation study of early childhood programs. Final Report Volume II: Chapter 1-Funded Early Childhood Programs; 1993. Available: http://files.eric.ed.gov/fulltext/ED366469.pdf. Accessed July 3, 2015.

17. Travers J, Goodson BD, Singer JD, Connell DB. Research results of the National Day Care Study. Final Report of the National Day Care Study. Volume II. Cambridge, MA: Abt Associates, Inc; 1980. Available: http://files.eric.ed.gov/fulltext/ED195336.pdf.

18. Zellman G, Perlman M, Le V, Setodji C. Assessing the validity of the Qualistar Early Learning Quality Rating and Improvement System as a tool for improving child-care quality. Santa Monica, CA: RAND Education; 2008. Available: http://www.rand.org/content/dam/rand/pubs/monographs/2008/RAND_MG650.pdf.

19. Zill N, Resnick G, Kim K, O'Donnell K, Sorongon A, Ziv Y, et al. Head Start Performance Measures Center Family and Child Experiences Survey (FACES 2000): Technical Report. Washington, DC: U.S. Department of Health and Human Services, Administration for Children and Families, Office of Planning, Research and Evaluation; 2006. Available: http://www.acf.hhs.gov/sites/default/files/opre/tech2k_final2.pdf.

20. Burchinal M, Cryer D, Clifford R, Howes C. Caregiver training and classroom quality in child care centers. Appl Dev Sci. 2002;6: 2-11. doi:10.1207/S1532480XADS0601_01.

21. Burchinal M, Nelson L. Family selection and child care experiences: implications for studies of child outcomes. Early Child Res Q. 2000;15: 385-411. doi:10.1016/S0885-2006(00)00072-7.

22. Mashburn A, Justice L, Downer J, Pianta R. Peer effects on children's language achievement during pre-kindergarten. Child Dev. 2009;80: 686-702. doi:10.1111/j.1467-8624.2009.01291.x.

23. McCartney K. Effect of quality of day care environment on children's language development. Dev Psychol. 1984;20: 244-260. doi:10.1037/0012-1649.20.2.244.

24. Studer M. Quality of center care and preschool cognitive outcomes: differences by family income. Sociol Stud Child Dev. 1992;5: 49-72.

25. Zill N, Resnick G, Kim K, O'Donnell K, Sorongon A, McKey RH, et al. Head Start FACES 2000: A Whole Child Perspective on Program Performance. Washington DC, US: U.S. Department of Health and Human Services, Administration for Children and Families, Office of Planning, Research and Evaluation; 2003:1-160. Available: http://www.acf.hhs.gov/sites/default/files/opre/faces00_4thprogress.pdf.

26. Clarke-Stewart K, Lee A, Allhusent V, Kim M, McDowell D. Observed differences between early childhood programs in the U.S. and Korea: reflections of "developmentally appropriate practices" in two cultural contexts. J Appl Dev Psychol. 2006;27: 427-443. doi:10.1016/j.appdev.2006.06.006.

27. Holloway S, Reichhart-Erickson M. The relationship of day care quality to children's free-play behavior and social problem-solving skills. Early Child Res Q. 1988;3: 39-53. doi:10.1016/0885-2006(88)90028-2.

28. Holloway S, Reichart-Erickson M. Child-care quality, family structure, and maternal expectations: relationship to preschool children's peer relations. J Appl Dev Psychol. 1989;10: 281-298. doi:10.1016/0193-3973(89)90031-2.

29. Love J, Ryer P, Faddis B. Caring environments: program quality in California's publicly funded child development programs: report on the legislatively mandated 1990-91 staff/child ratio Study. Portsmouth, NH: RMC Research Corporation; 1992.
