# Supplementary material for: Child-Staff Ratios in Early Childhood Education and Care Settings and Child Outcomes: A Systematic Review and Meta-Analysis
Source: PLoS One. 2017 Jan 19;12(1):e0170256. doi: 10.1371/journal.pone.0170256 (PMC5245988; doi:10.1371/journal.pone.0170256)
Supplement: S4 File — (PDF) [file pone.0170256.s004.pdf]

# Child-Staff Ratios in Early Childhood Education and Care Settings and Child Outcomes: A Systematic Review and Meta-Analysis

## Supplemental Information 4

Child-Staff Ratio Quality Indicator: List of Child Outcome Variables

| Outcome Category | Number of Measures | Child Outcome Variable | Number of Studies | Number of Samples |
|---|---|---|---|---|
| **Approach** | n=5 | Child Behavior Inventory - Creativity (CBI) | 1 | 1 |
| | | Child Behavior Inventory - Dependence (CBI) | 2 | 2 |
| | | Child Behavior Inventory - Dependence (Parent) (CBI) | | |
| | | Child Behavior Inventory - Dependence (Teacher) (CBI) | | |
| | | Child Behavior Inventory - Distractibility (CBI) | 1 | 1 |
| | | Child Behavior Inventory - Independent (CBI) | 1 | 1 |
| | | Child Behavior Inventory - Task Orientation (CBI) | 2 | 2 |
| | | Child Behavior Inventory - Task Orientation (Parent) (CBI) | | |
| | | Child Behavior Inventory - Task Orientation (Teacher) (CBI) | | |
| **Cognitive** | n=7 | Bayley Scales of Infant Development-R: Mental Development Index | 1 | 1 |
| | | Bracken Basic Concept Scale - School Readiness (BBCS) | 2 | 2 |
| | | Child Behavior Inventory - Intelligence (CBI) | 3 | 3 |
| | | Child Behavior Inventory - Intelligence (Parent) (CBI) | | |
| | | Child Behavior Inventory - Intelligence (Teacher) (CBI) | | |
| | | Identifying Colors (Color Naming) | 1 | 1 |
| | | Intellectual Ability | 1 | 1 |
| | | Preschool Inventory (PSI) | 3 | 3 |
| | | FACES - Social Awareness in the classroom | 1 | 1 |
| **Combo** | n=1 | Child Behavior Rating Scale (CBRS) | 1 | 1 |
| **Language** | n=19 | Academic Rating Scale: Language & Literacy | 3 | 4 |
| | | Adaptive Language Inventory (ALI) | 1 | 1 |
| | | Communication task | 1 | 1 |
| | | Early Childhood Longitudinal Study - Literacy (ECLS) | 1 | 1 |
| | | Identifying Letters (Letter-Naming Test, Naming Letters) | 5 | 6 |
| | | Oral & Written Language Scales - Oral Expression Scale (OWLS) | 5 | 5 |
| | | Oral & Written Language Scales - Total Scale (OWLS) | 1 | 1 |
| | | Peabody Picture Vocabulary Test (PPVT-III) | 16 | 16 |

| Child-Staff Ratio Quality Indicator: List of Child Outcome Variables | | | | |
|---|---|---|---|---|
| **Outcome Category** | **Number of Measures** | **Child Outcome Variable** | **Number of Studies** | **Number of Samples** |
| | | Peabody Picture Vocabulary Test (PPVT-R) | | |
| | | Peabody Picture Vocabulary Test (TVIP) | | |
| | | Preschool Language Assessment Instrument (PLAI) | 1 | 1 |
| | | Reynell Developmental Language Scales (RDLS) - Expressive Language | 1 | 1 |
| | | Reynell Developmental Language Scales (RDLS) - Language Comprehension | 1 | 1 |
| | | Sequenced Inventory of Communication Development-Revised (SICD-R) -Expressive Communication Age | 1 | 1 |
| | | Sequenced Inventory of Communication Development-Revised (SICD-R)-Receptive Communication Age | 1 | 1 |
| | | Story & Print Concepts - Book Knowledge | 1 | 1 |
| | | Woodcock Johnson - Dictation (WJ-R) | 1 | 1 |
| | | Woodcock Johnson - Letter Word Identification (WJ-III) | 4 | 4 |
| | | Woodcock Johnson - Letter Word Identification (WJ-R) | | |
| | | Woodcock Muñoz - Indentificación de Letras y Palabras | | |
| | | Woodcock Johnson - Passage Comprehension (WJ-III) | 1 | 1 |
| | | Woodcock Johnson – Revised Reading | 1 | 1 |
| | | Woodcock Johnson - Rhyming (WJ-III R) | 3 | 3 |
| **Math** | n=5 | Counting Task (One-One Counting) | 1 | 1 |
| | | Early Childhood Longitudinal Study - Math (ECLS) | 1 | 1 |
| | | K-ABC (German Version) Arithmetic (K-ABC) | 1 | 1 |
| | | Identifying Numbers (Naming Numbers) | 1 | 1 |
| | | Woodcock Johnson - Applied Problems (WJ-III) | 9 | 10 |
| | | Woodcock Johnson - Applied Problems (WJ-) | | |
| | | Woodcock Mu˜noz – Problemas | | |
| **Physical** | n=1 | Design Copying | 1 | 1 |
| **Positive Behavior** | n=22 | Adaptive Social Behavior Inventory – Comply | 1 | 1 |
| | | Adaptive Social Behavior Inventory - Comply (Parent) (ASBI) | | |
| | | Adaptive Social Behavior Inventory - Comply (Teacher) (ASBI) | | |
| | | Adaptive Social Behavior Inventory – Express | 1 | 1 |
| | | Adaptive Social Behavior Inventory - Express | | |

| Child-Staff Ratio Quality Indicator: List of Child Outcome Variables | | | | |
|---|---|---|---|---|
| **Outcome Category** | **Number of Measures** | **Child Outcome Variable** | **Number of Studies** | **Number of Samples** |
| | | (Parent) (ASBI) | | |
| | | Adaptive Social Behavior Inventory - Express (Teacher) (ASBI) | | |
| | | Child Behavior Inventory - Considerateness (CBI) | 2 | 2 |
| | | Child Behavior Inventory - Considerateness (Parent) (CBI) | | |
| | | Child Behavior Inventory - Considerateness (Teacher) (CBI) | | |
| | | Child Behavior Inventory - Sociability (CBI) | 2 | 2 |
| | | Child Behavior Inventory - Sociability (Parent) (CBI) | | |
| | | Child Behavior Inventory - Sociability (Teacher) (CBI) | | |
| | | Cooperative Behavior | 1 | 1 |
| | | Early Childhood Longitudinal Study - Attention & Concentration | 1 | 1 |
| | | Early Childhood Longitudinal Study - Attention & Concentration (Teacher) | | |
| | | Early Childhood Longitudinal Study - Attention & Concentration (Parent) | | |
| | | Early Childhood Longitudinal Study - Emotional & Behavioral Regulation | 1 | 1 |
| | | Early Childhood Longitudinal Study - Emotional & Behavioral Regulation (Teacher) | | |
| | | Early Childhood Longitudinal Study - Emotional & Behavioral Regulation (Parent) | | |
| | | Early Childhood Longitudinal Study - Prosocial | 1 | 1 |
| | | Early Childhood Longitudinal Study – Prosocial (Teacher) | | |
| | | Early Childhood Longitudinal Study – Prosocial (Parent) | | |
| | | Observational Record of the Caregiving Environment – Self-Reliance (ORCE) | 1 | 1 |
| | | Observational Record of the Caregiving Environment - Sustained Attention (ORCE) | 1 | 1 |
| | | Positive Social Behavior Composite | 1 | 1 |
| | | Social - Cognitive Ability | 1 | 1 |
| | | Social - Competence with Stranger | 1 | 1 |
| | | Social - Competence with Visitor | 1 | 1 |
| | | Social Competence (rated by teacher) | 1 | 1 |
| | | Social Problem-Solving Skills Procedure - Prosocial Categories | 1 | 1 |

| Child-Staff Ratio Quality Indicator: List of Child Outcome Variables | | | | |
|---|---|---|---|---|
| **Outcome Category** | **Number of Measures** | **Child Outcome Variable** | **Number of Studies** | **Number of Samples** |
| | | Social Problem-Solving Skills Procedure - Prosocial Responses | 2 | 2 |
| | | Social Skills Rating Scale | 1 | 1 |
| | | Social Skills Rating Scale - Parent | | |
| | | Social Skills Rating Scale - Teacher | | |
| | | Social Skills and Behavior Problems Scale - Social Competence (SSBS) | 1 | 1 |
| | | Student-Teacher Relationship Scale - shortened (STRS) | 1 | 1 |
| | | Teacher Child Rating Scale - Social Competence (TCRS) | 4 | 5 |
| | | The California Preschool Social Competency Scale Social Competence | 1 | 1 |
| **Problem Behavior** | n=23 | Behavior Problems Composite | 1 | 1 |
| | | Behavior Problems Index - Antisocial (BPI) | 1 | 1 |
| | | Behavior Problems Index - Attention-Deficit (BPI) | 1 | 1 |
| | | Behavior Problems Index - Depressed (BPI) | 1 | 1 |
| | | Behavior Problems Index - Immature/Dependent (BPI) | 1 | 1 |
| | | Child Behavior Checklist - Externalizing (Parent) (CBCL) | 1 | 1 |
| | | Child Behavior Checklist - Internalizing (Parent) (CBCL) | 1 | 1 |
| | | Child Behavior Inventory - Apathy (CBI) | 1 | 1 |
| | | Child Behavior Inventory - Behavior Problems (CBI) | 1 | 1 |
| | | Child Behavior Inventory - Hostility (CBI) | 1 | 1 |
| | | FACES - Aggressive Behavior | 1 | 1 |
| | | FACES - Aggressive Behavior (Parent) | | |
| | | FACES - Aggressive Behavior (Teacher) | | |
| | | FACES - Hyperactive Behavior | 1 | 1 |
| | | FACES - Hyperactive Behavior- Parent | | |
| | | FACES - Hyperactive Behavior- Teacher | | |
| | | FACES - Withdrawn Behaviors | 1 | 1 |
| | | FACES - Withdrawn Behaviors - Parent | | |
| | | FACES - Withdrawn Behaviors - Teacher | | |
| | | Preschool Behavior Questionnaire - Aggression | 1 | 1 |
| | | Preschool Behavior Questionnaire - Aggression (PBQ) (Parent) | | |
| | | Preschool Behavior Questionnaire - Aggression ( (PBQ) (Teacher) | | |

| Child-Staff Ratio Quality Indicator: List of Child Outcome Variables | | | | |
|---|---|---|---|---|
| **Outcome Category** | **Number of Measures** | **Child Outcome Variable** | **Number of Studies** | **Number of Samples** |
| | | Preschool Behavior Questionnaire - Anxiety (PBQ) | 1 | 1 |
| | | Preschool Behavior Questionnaire - Anxiety (PBQ) (Parent) | | |
| | | Preschool Behavior Questionnaire - Anxiety (PBQ) (Teacher) | | |
| | | Preschool Behavior Questionnaire - Hyperactivity (PBQ) | 2 | 2 |
| | | Preschool Behavior Questionnaire – Hyperactivity/Distractible (PBQ) | | |
| | | Preschool Behavior Questionnaire - Hyperactivity (PBQ) (Parent) | | |
| | | Preschool Behavior Questionnaire - Hyperactivity (PBQ) Teacher) | | |
| | | Preschool Behavior Scale - Hyperactivity (PBS) | 1 | 1 |
| | | Social Problem-Solving Skills Procedure - Antisocial Categories | 1 | 1 |
| | | Social Problem-Solving Skills Procedure - Antisocial Responses | 1 | 1 |
| | | Social Skills and Behavior Problems Scale - Behavior Problems (SSBS) | 1 | 1 |
| | | Teacher Child Rating Scale - Behavior Problems (TCRS) | 3 | 4 |
| | | Teacher's Report Form - Externalizing (Teacher) (TCRF) | 2 | 2 |
| | | Teacher's Report Form - Internalizing (Teacher) (TCRF) | 2 | 2 |
| **Total** | **n=83** | | | |
