# Supplementary material for: Child-Staff Ratios in Early Childhood Education and Care Settings and Child Outcomes: A Systematic Review and Meta-Analysis
Source: PLoS One. 2017 Jan 19;12(1):e0170256. doi: 10.1371/journal.pone.0170256 (PMC5245988; doi:10.1371/journal.pone.0170256)
Supplement: S5 File — (PDF) [file pone.0170256.s005.pdf]

Child-Staff Ratios in Early Childhood Education and Care Settings and Child Outcomes: A Systematic Review and Meta-Analysis

Supplemental Information 5

List of Acronyms Used in the Description of Studies	
**Acronym Category**	**Full Name**
**Journals**	American Journal of Public Health (AJPH)<br>Applied Developmental Science (ADS)<br>Child Development (CD)<br>Developmental Psychology (DP)<br>Early Child Development & Care (ECDC)<br>Early Childhood Research Quarterly (ECRQ)<br>Early Education & Development (EED)<br>Journal of Applied Developmental Psychology (JADP)<br>Merrill-Palmer Quarterly (MPQ)<br>Sociological Studies of Child Development (SSCD)
**Large Sample**	California Staff/Child Ratio Study (CSRS)<br>Cost, Quality and Outcomes Study (CQO)<br>Early Childhood Longitudinal Study-Birth Cohort (ECLS-B)<br>Head Start Family and Children Experiences Study (FACES)<br>National Center for Early Development and Learning Multi-State Study of Kindergarten & National Day Care Study (NDSC)<br>National Institute of Child Health and Human Development (NICHD)<br>National Longitudinal Survey of Labor Market Experience of Youth (NLSY)<br>Observational Study of Early Childhood Programs (OSECP)<br>Quality Rating and Improvement Systems - Colorado (QRIS)<br>Study of State-Wide Early Education Programs (NCEDL & SWEEP)
**Covariates**	Associate Degree (AA)<br>Authoritative Parenting Style (AP)<br>Bachelor's Degree (BA)<br>Bachelor's Science (BS)<br>Caregiver Interaction Scale (CIS)<br>Child Development (CD)<br>Child Development Certificate (CDC)<br>Classroom Assessment Scoring System (CLASS)<br>Developmentally Appropriate Beliefs (DAP)<br>Dual Language Learner (DLL)<br>Early Childhood Education (ECE)<br>English Language Learner (ELL)<br>Environment Rating Scales (ECERS)<br>Home Learning Environment (HLE)<br>Individualized Educational Plan (IEP)<br>Socio-Economic Status (SES)<br>Special Supplemental Nutrition Program for Women, Infants and Children (WIC)<br>Teacher-Child (T-C)<br>Temporary Assistance for Needy Families (TANF)
